# Implications of Racial Differences in the Shifts in the Setting of Care for Alzheimer’s Disease and Related Dementias

**DOI:** 10.1093/geroni/igab046.2438

**Published:** 2021-12-17

**Authors:** Arseniy Yashkin, Galina Gorbunova, Anatoliy Yashin, Igor Akushevich

**Affiliations:** 1 Duke University, Morrisville, North Carolina, United States; 2 Duke University, Durham, North Carolina, United States

## Abstract

The prevailing setting of care has strong associations with the progression of a disease at time of first diagnosis, subsequent treatment, resulting health outcomes as well as both long-term and short-term costs. The care of Alzheimer’s Disease (AD) and Related Dementias (ADRD) has been experiencing a shift from skilled nursing facility to home health care. However, changes in practice do not disseminate equally across the race/ethnicity spectrum of the U.S. and disadvantaged race/ethnicity-related groups often encounter differing conditions from those experienced by the majority. In this study, we calculated the race/ethnicity-related direct healthcare costs of individuals with AD and ADRD, stratified by care-provider structure (physician, inpatient, outpatient, skilled nursing facility, home health, hospice), and modeled the trends and the relative contributions of each setting over the 1991-2017 period using administrative claims from a 5% sample of Medicare beneficiaries. Inflation and the gradual switch of Medicare compensation to the HCC model between 2004 and 2007 were accounted for. We then applied an inverse probability weighting algorithm to propensity-score-match the AD/ADRD race/ethnicity-specific groups to Medicare beneficiaries to make them comparable in demographics and co-morbidity status but without AD/ADRD. Finally, we performed a comparison of the Medicare costs and associated survival within (AD/ADRD vs. No AD/ADRD) and between (Black vs. White vs. Hispanic) race/ethnicity-related groups. Comparisons were done for: i)1-year before; ii) 1-year after iii) years 2-11; iv)years 12-21 and v) years 22+ after an AD/ADRD diagnosis. We found significant race/ethnicity-related differences in costs and survival both before and after propensity score matching.

